# GrabCut-Based Human Segmentation in Video Sequences

**DOI:** 10.3390/s121115376

**Published:** 2012-11-09

**Authors:** Antonio Hernández-Vela, Miguel Reyes, Víctor Ponce, Sergio Escalera

**Affiliations:** 1 Departamento MAIA, Universitat de Barcelona, Gran Via 585, 08007 Barcelona, Spain; E-Mails: mreyes@cvc.uab.cat (M.R.); vponce@cvc.uab.cat (V.P.); sergio@maia.ub.es (S.E.); 2 Centre de Visió per Computador, Campus UAB, Edifici O, 08193 Bellaterra, Barcelona, Spain

**Keywords:** segmentation, human pose recovery, GrabCut, GraphCut, Active Appearance Models, Conditional Random Field

## Abstract

In this paper, we present a fully-automatic Spatio-Temporal GrabCut human segmentation methodology that combines tracking and segmentation. GrabCut initialization is performed by a HOG-based subject detection, face detection, and skin color model. Spatial information is included by Mean Shift clustering whereas temporal coherence is considered by the historical of Gaussian Mixture Models. Moreover, full face and pose recovery is obtained by combining human segmentation with Active Appearance Models and Conditional Random Fields. Results over public datasets and in a new Human Limb dataset show a robust segmentation and recovery of both face and pose using the presented methodology.

## 1. Introduction

Human segmentation in uncontrolled environments is a hard task because of the constant changes produced in natural scenes: illumination changes, moving objects, changes in the point of view, occlusions, just to mention a few. Because of the nature of the problem, a common way to proceed is to discard most part of the image so that the analysis can be performed on a reduced set of small candidate regions. In [1], the authors propose a full-body detector based on a cascade of classifiers [2] using HOG features. This methodology is currently being used in several works related to the pedestrian detection problem [3]. GrabCut [4] has also shown high robustness in Computer Vision segmentation problems, defining the pixels of the image as nodes of a graph and extracting foreground pixels via iterated Graph Cut optimization. This methodology has been applied to the problem of human body segmentation with high success [5,6]. In the case of working with sequences of images, this optimization problem can also be considered to have temporal coherence. In the work of [7], the authors extended the Gaussian Mixture Model (GMM) of GrabCut algorithm so that the color space is complemented with the derivative in time of pixel intensities in order to include temporal information in the segmentation optimization process. However, the main problem of that method is that moving pixels corresponds to the boundaries between foreground and background regions, and thus, there is no clear discrimination.

Once a region of interest is determined, pose is often recovered by the determination of the body limbs together with their spatial coherence (also with temporal coherence in case of image sequences). Most of these approaches are probabilistic, and features are usually based on edges or “appearance”. In [8], the author propose a probabilistic approach for limb detection based on edge learning complemented with color information. The image of probabilities is then formulated in a Conditional Random Field (CRF) scheme and optimized using belief propagation. This work has obtained robust results and has been extended by other authors including local GrabCut segmentation and temporal refinement of the CRF model [5,6].

In this paper, we propose a full-automatic Spatio-Temporal GrabCut human segmentation methodology, which benefits from the combination of tracking and segmentation. First, subjects are detected by means of a HOG-based cascade of classifiers. Face detection and skin color model are used to define a set of seeds used to initialize GrabCut algorithm. Spatial information is taken into account by means of Mean Shift clustering, whereas temporal information is considered taking into account the pixel probability membership to an historical of Gaussian Mixture Models. Moreover, the methodology is combined with Shape and Active Appearance Models (AAM) to define three different meshes of the face, one near frontal view, and the other ones near lateral views. Temporal coherence and fitting cost are considered in conjunction with GrabCut segmentation to allow a smooth and robust face fitting in video sequences. Finally, the limb detection and a CRF model are applied on the obtained segmentation, showing high robustness capturing body limbs due to the accurate human segmentation. The main limitation of our approach is that it depends on a correct detection of the person and his/her face, in order to get the desired result. In order to test the proposed methodology, we use public datasets and present a new Human Limb dataset useful for human segmentation, limb detection, and pose recovery purposes.

The rest of the paper is organized as follows: Section 2 describes the proposed methodology, presenting the spatio-temporal GrabCut segmentation, the AAM for face fitting, and the pose recovery methodology. Experimental results on public and novel datasets are performed in Section 3. Finally, Section 4 concludes the paper.

## 2. Full-Body Pose Recovery

**Figure 1.**
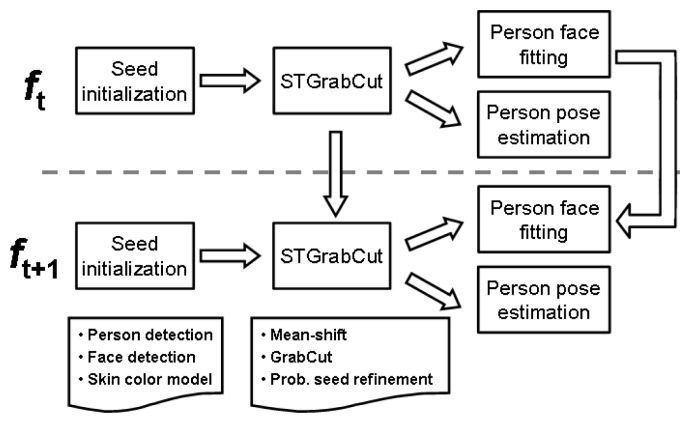
Overall block diagram of the methodology.

In this section, we present the Spatio-Temporal GrabCut methodology to deal with the problem of automatic human segmentation in video sequences. Then, we describe the Active Appearance Models used to recover the face, and the body pose recovery methodology based on the approach of [8]. All methods presented in this section are combined to improve final segmentation and pose recovery. Figure 1 illustrates the different modules of the project.

### 2.1. GrabCut Segmentation

In [4], the authors proposed an approach to find a binary segmentation(background and foreground) of an image by formulating an energy minimization scheme as the one presented in [9–11], extended using color instead of just gray-scale information. Given a color image *I*, let us consider the array *z* = (*z*_1_, …, *z_n_*, …, *z_N_*) of *N* pixels where *z_i_* = (*R_i_*, *G_i_*, *B_i_*), *i* ∈ [1, …, *N*] in RGB space. The segmentation is defined as array ***α*** = (*α*_1_, …*α_N_*), *α_i_* ∈ {0, 1}, assigning a label to each pixel of the image indicating if it belongs to background or foreground. A trimap *T* is defined by the user—in a semi-automatic way—consisting of three regions: *T_B_*, *T_F_* and *T_U_*, each one containing initial background, foreground, and uncertain pixels, respectively. Pixels belonging to *T_B_* and *T_F_* are clamped as background and foreground respectively—which means GrabCut will not be able to modify these labels, whereas those belonging to *T_U_* are actually the ones the algorithm will be able to label. Color information is introduced by GMMs. A full covariance GMM of *K* components is defined for background pixels (*α_i_* = 0), and another one for foreground pixels (*α_j_* = 1), parametrized as follows

(1) θ={π(α,k),μ(α,k),∑(α,k),α∈{0,1},k=1‥K},

being *π* the weights, *μ* the means and Σ the covariance matrices of the model. We also consider the array **k** = {*k*_1_, …, *k_i_*, …*k_N_*}, *k_i_* ∈ {1, …*K*}, *i* ∈ [1, …, *N*] indicating the component of the background or foreground GMM (according to *α_i_*) the pixel *z_i_* belongs to. The energy function for segmentation is then

(2) E(α,k,θ,z)=U(α,k,θ,z)+V(α,z),

where **U** is the likelihood potential, based on the probability distributions *p*(·) of the GMM:

(3) $$U(\alpha ,k,\theta ,z)=\sum_{i}-\log p(z_{i}|\alpha_{i},k_{i},\theta )-\log\pi (\alpha_{i},k_{i})$$

and *V* is a regularizing prior assuming that segmented regions should be coherent in terms of color, taking into account a neighborhood *C* around each pixel

(4) $$V(\alpha ,z)=\gamma \sum_{\{m,n\}\in C}\left[\alpha_{n}\neq \alpha_{m}]\exp(-\beta {\left\|z_{m}-z_{n}\right\|}^{2}\right)$$

With this energy minimization scheme and given the initial trimap *T*, the final segmentation is performed using a minimum cut algorithm [9,10,12]. The classical semi-automatic GrabCut algorithm is summarized in Algorithm 1.

**Algorithm 1 Original GrabCut algorithm.**
 1:Trimap *T* initialization with manual annotation. 2:Initialize *α_i_* = 0 for *i* ∈ *T_B_* and *α_i_* = 1 for *i* ∈ *T_U_* ∪ *T_F_*. 3:Initialize Background and Foreground GMMs from sets *α_i_* = 0 and *α*_i_ = 1 respectively, with *k*-means. 4:Assign GMM components to pixels. 5:Learn GMM parameters from data z. 6:Estimate segmentation: Graph-cuts. 7:Repeat from step 4, until convergence.


### 2.2. Automatic Initialization

**Figure 2.**
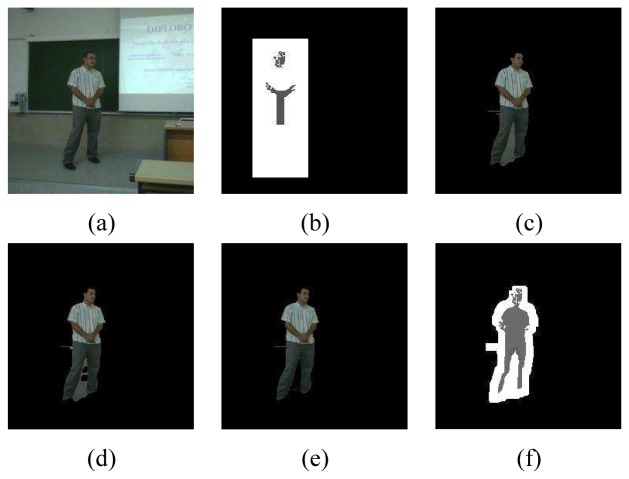
STGrabcut pipeline example: (**a**) Original frame, (**b**) Seed initialization, (**c**) GrabCut, (**d**) Probabilistic re-assignment, (**e**) Refinement and (**f**) Initialization mask for *f_t_*_+1_

Our proposal is based on the previous GrabCut framework, focusing on human body segmentation, being fully automatic, and extending it by taking into account temporal coherence. We refer to each frame of the video as *f_t_*, *t* ∈ {1, …, *M*} being *M* the length of the sequence. Given a frame *f_t_*, we first apply a person detector based on a cascade of classifiers using HOG features [1]. Then, we initialize the trimap *T* from the bounding box *B* retuned by the detector: *T_U_* = {*z_i_* ∈ *B*}, *T_B_* = {*z_i_* ∉ *B*}. Furthermore, in order to increase the accuracy of the segmentation algorithm, we include Foreground seeds exploiting spatial and appearance prior information. On one hand, we define a small central rectangular region *R* inside *B*, proportional to *B* in such a way that we are sure it corresponds to the person. Thus, pixels inside *R* are set to foreground. On the other, we apply a face detector based on a cascade of classifiers using Haar-like features [2] over *B*, and learn a skin color model *h_skin_* consisting of a histogram over the *Hue* channel of the *HSV* image representation. All pixels inside *B* fitting in *h_skin_* are also set to foreground. Therefore, we initialize *T_F_* = {*z_i_* ∈ *R*} ∪ {*z_i_* ∈ *δ*(*z_i_*, *h_skin_*)}, where *δ* returns the set of pixels belonging to the color model defined by *h_skin_*. An example of seed initialization is shown in Figure 2(b).

### 2.3. Spatial Extension

Once we have initialized the trimap, we can apply the iterative minimization algorithm shown in steps 4 to 7 of original GrabCut (Algorithm 1). However, instead of applying k-means for the initialization of the GMMs we propose to use Mean-Shift clustering, which also takes into account spatial coherence. Given an initial estimation of the distribution modes *m_h_*(**x**^0^) and a kernel function *g*, Mean-shift iteratively updates the mean-shift vector with the following formula:

(5) $$m_{h}(x)=\frac{\sum_{i=1}^{n}x_{i}g\left({\left\|\frac{x-x_{i}}{h}\right\|}^{2}\right)}{\sum_{i=1}^{n}g\left({\left\|\frac{x-x_{i}}{h}\right\|}^{2}\right)}$$

until it converges, where **x***_i_* contains the value of pixel *z_i_* in CIELuv space and its spatial coordinates, and returns the centers of the clusters (distribution modes) found. After convergence, we obtain a segmentation ***α****^t^* and the updated foreground and background GMMs ***θ****^t^* at frame *f_t_*, which are used for further initialization at frame *f_t_*_+1_. The result of this step is shown in Figure 2(c). Finally, we refine the segmentation of frame *f_t_* eliminating false positive foreground pixels. By definition of the energy minimization scheme, GrabCut tends to find convex segmentation masks having a lower perimeter, given that each pixel on the boundary of the segmentation mask contributes on the global cost. Therefore, in order to eliminate these background pixels (commonly in concave regions) from the foreground segmentation, we re-initialize the trimap *T* as follows

(6) $$\begin{array}{lll} T_{B} & = & \{z_{i}|\alpha_{i}=0\}\cup \left\{z_{i}|\frac{\sum_{k=t-j}^{t}p(z_{i}|\alpha_{i}=0,k_{i},\theta^{k})}{j}>\frac{\sum_{k=t-j}^{t}p(z_{i}|\alpha_{i}=1,k_{i},\theta^{k})}{j}\right\}\\ T_{F} & = & \left\{z_{i}\in \delta (z_{i},h_{\text{skin}})\right\}\\ T_{U} & = & \{z_{i}|\alpha_{i}=1\}\backslash T_{B}\backslash T_{F} \end{array}$$

where the pixel background probability membership is computed using the GMM models of previous *j* segmentations. This formulation can also be extended to detect false negatives. However, in our case we focus on false positives since they appear frequently in the case of human segmentation. The result of this step is shown in Figure 2(d). Once the trimap has been redefined, false positive foreground pixels still remain, so the new set of seeds is used to iterate again GrabCut algorithm, resulting in a more accurate segmentation, as we can see in Figure 2(e).

### 2.4. Temporal Extension

Considering *A* as the binary image representing ***α*** at *f_t_* (the one obtained before the refinement), we initialize the trimap for *f_t_*_+1_ as follows

(7) $$\begin{array}{lll} T_{F} & = & \left\{z_{i}\in I|z_{i}\in A⊖ST_{e},\alpha (z_{i})=1\right\}\\ T_{U} & = & \{z_{i}\in I|z_{i}\in A⊕ST_{d},\alpha (z_{i})=1\}\backslash T_{F}\\ T_{B} & = & \left\{z_{i},z_{i}\in I\}\backslash (T_{F}\cup T_{U}\right) \end{array}$$

where ⊖ and ⊕ are erosion and dilation operations with their respective structuring elements *ST_e_* and *ST_d_*, *α_i_* := *α*(*z_i_*), and \ represents the set difference operation. The structuring elements are simple squares of a given size depending on the size of the person and the degree of movement we allow from *f_t_* to *f_t_*_+1_, assuming smoothness in the movement of the person. An example of a morphological mask is shown in Figure 2(f). Spatial information could be also included in the mean-shift algorithm in conjunction with color and spatial information. However, we included this information explicitly to be anisotropic. The whole segmentation methodology is detailed in the ST-GrabCut Algorithm 2.

**Algorithm 2 Spatio-Temporal GrabCut algorithm.**
 1:Person detection on *f*_1_. 2:Face detection and skin color model learning. 3:Trimap *T* initialization with detected bounding box and learnt skin color model. 4:Initialize *α_i_* = 0 for *i* ∈ *T_B_* and *α_i_* = 1 for *i* ∈ *T_U_* ∪ *T_F_*. 5:Initialize Background and Foreground GMMs from sets *α_i_* = 0 and *α_i_* = 1 respectively, with Mean-shift. 6:**for**
*t* = 1 … *M* 7: Person detection on *f_t_*. 8: Assign GMM components to pixels of *f_t_*. 9: Learn GMM parameters from data z. 10: Estimate segmentation: Graph-cuts. 11: Repeat from step 8, until convergence. 12: Re-initialize trimap *T* (Equation (6)). 13: Assign GMM components to pixels. 14: Learn GMM parameters from data z. 15: Estimate segmentation: Graph-cuts. 16: Repeat from step 12, until convergence. 17: Initialize trimap *T* using segmentation obtained in step 11 after convergence (equation 7) for *f_t_*_+1_. 18:**end for**


### 2.5. Face Fitting

Once we have properly segmented the body region, the next step consists of fitting the face and the body limbs. For the case of face recovery, we base our procedure on mesh fitting using AAM, combining Active Shape Models and color and texture information [13].

AAM is generated by combining a model of shape and texture variation. First, a set of points are marked on the face of the training images that are aligned, and a statistical shape model is build [14]. Each training image is warped so the points match those of the mean shape. This is raster scanned into a texture vector, **g**, which is normalized by applying a linear transformation, **g** ↦ (**g** − *μ_g_***1**)/*σ_g_*, where **1** is a vector of ones, and *μ_g_* and *σ_g_* are the mean and variance of elements of **g**. After normalization, **g***^T^***1** = 0 and |**g**| = 1. Then, principal component analysis is applied to build a texture model. Finally, the correlations between shape and texture are learnt to generate a combined appearance model. The appearance model has parameter **c** controlling the shape and texture according to

(8) $$x=\bar{x}+Q_{s}c$$

(9) $$g=\bar{g}+Q_{g}c$$

where *x̄* is the mean shape, *ḡ* the mean texture in a mean shaped patch, and **Q***_s_*, **Q***_g_* are matrices designing the modes of variation derived from the training set. A shape **X** in the image frame can be generated by applying a suitable transformation to the points, **x : X** = *S_t_*(**x**). Typically, *S_t_* will be a similarity transformation described by a scaling *s*, an in-plane rotation, *θ*, and a translation (*t_x_*, *t_y_*).

Once constructed the AAM, it is deformed on the image to detect and segment the face appearance as follows. During matching, we sample the pixels in the region of interest ***g****_im_* = *T_u_*(**g**) = (*u*_1_ + 1)**g***_im_* + *u*_2_**1**, where **u** is the vector of transformation parameters, and project into the texture model frame, $g_{s}=T_{u}^{-1}(g_{im})$. The current model texture is given by **g***_m_* = *ḡ* + **Q***_g_***c**, and the difference between model and image (measured in the normalized texture frame) is as follows

(10) $$r(p)=g_{s}-g_{m}$$

Given the error *E* = |**r**|^2^, we compute the predicted displacements *δ***p** = −**Rr**(**p**), where $R={\left(\frac{\partial r^{T}}{\partial p}\frac{\partial r}{\partial p}\right)}^{-1}\frac{\partial r^{T}}{\partial p}$. The model parameters are updated **p** ↦ **p** + *kδ***p**, where initially *k* = 1. The new points **X**′ and model frame texture $g_{m}^{\prime }$ are estimated, and the image is sampled at the new points to obtain $g_{mi}^{\prime }$ and the new error vector $r\prime =T_{u\prime }^{-1}(g_{im}^{\prime })-g_{m}^{\prime }$. A final condition guides the end of each iteration: if |**r**′|^2^ < *E*, then we accept the new estimate, otherwise, we set to *k* = 0.5, *k* = 0.25, and so on. The procedure is repeated until no improvement is made to the error.

With the purpose to discretize the head pose between frontal face and profile face, we create three AAM models corresponding to the frontal, right, and left view. Aligning every mesh of the model, we obtain the mean of the model. Finally, to determine the class of a fitted face by AAM models, that is given by its proximity to the closest mean model.

Taking into account the discontinuity that appears when a face moves from frontal to profile view, we use three different AAM corresponding to three meshes of 21 points: frontal view ℑ*_F_*, right lateral view ℑ*_R_*, and left lateral view ℑ*_L_*. In order to include temporal and spatial coherence, meshes at frame *f_t_*_+1_ are initialized by the fitted mesh points at frame *f_t_*. Additionally, we include a temporal change-mesh control procedure, as follows

(11) $${ℑ}^{t+1}=\min_{{ℑ}^{t+1}}\{E_{{ℑ}_{F}},E_{ℑR},E_{ℑL}\},{ℑ}^{t+1}\in \nu ({ℑ}^{t})$$

where *ν*(ℑ*^t^*) corresponds to the meshes contiguous to the mesh ℑ*^t^* fitted at time *t* (including the same mesh), and *E*_ℑ_*_i_* is the fitting error cost of mesh ℑ*_i_*. This constraint avoids false jumps and imposes smoothness in the temporal face behavior (e.g., a jump from right to left profile view is not allowed).

In order to obtain more accurate pose estimation, after fitting the mesh, we take advantage of its variability to differentiate among a set of head poses. Analyzing the spatial configuration of the 21 landmarks that composes a mesh, we create a new training set divided in five classes. We define five different head poses as follows: right, middle-right, frontal, middle-left, and left. In the training process, every mesh has been aligned, and PCA is applied to save the 20 most representative eigenvectors. Then, a new image is projected to that new space and classified to one of the five different head poses according to a 3-Nearest Neighbor rule.

**Figure 3.**
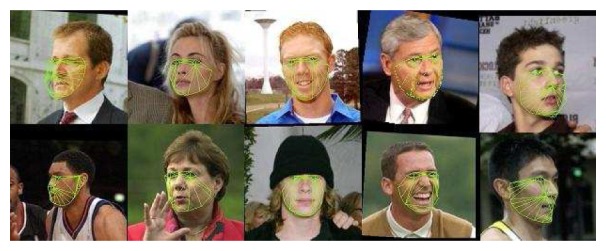
From left to right: left, middle-left, frontal, middle-right and right mesh fitting.

Figure 3 shows examples of the AAM model fitting and pose estimation in images (obtained from [15]) for the five different head poses.

### 2.6. Pose Recovery

Considering the refined segmented body region obtained using the proposed ST-GrabCut algorithm, we construct a pictorial structure model [16]. We use the method of Ramanan [6,8], which captures the appearance and spatial configuration of body parts. A person's body parts are tied together in a tree-structured conditional random field. Parts, *l_i_*, are oriented patches of fixed size, and their position is parameterized by location (*x*, *y*) and orientation *ϕ*. The posterior of a configuration of parts *L* = *l_i_* given a frame *f_t_* is

(12) $$P(L|f_{t})\propto \exp\left(\sum_{(i,j)\in E}\Psi (l_{i},l_{j})+\sum_{i}\Phi (l_{i}|f_{t})\right)$$

The pair-wise potential Ψ(*l_i_*, *l_j_*) corresponds to a spatial prior on the relative position of parts and embeds the kinematic constraints. The unary potential Φ(*l_i_*∣*I*) corresponds to the local image evidence for a part in a particular position. Inference is performed over tree-structured conditional random field.

Since the appearance of the parts is initially unknown, a first inference uses only edge features in Φ. This delivers soft estimates of body part positions, which are used to build appearance models of the parts and background (color histograms). Inference is then repeated with Φ using both edges and appearance. This parsing technique simultaneously estimates pose and appearance of parts. For each body part, parsing delivers a posterior marginal distribution over location and orientation (*x*, *y*, *ϕ*) [6,8].

## 3. Results

Before the presentation of the results, we discuss the data, methods and parameters of the comparative, and validation measurements.

### Data

**Figure 4.**
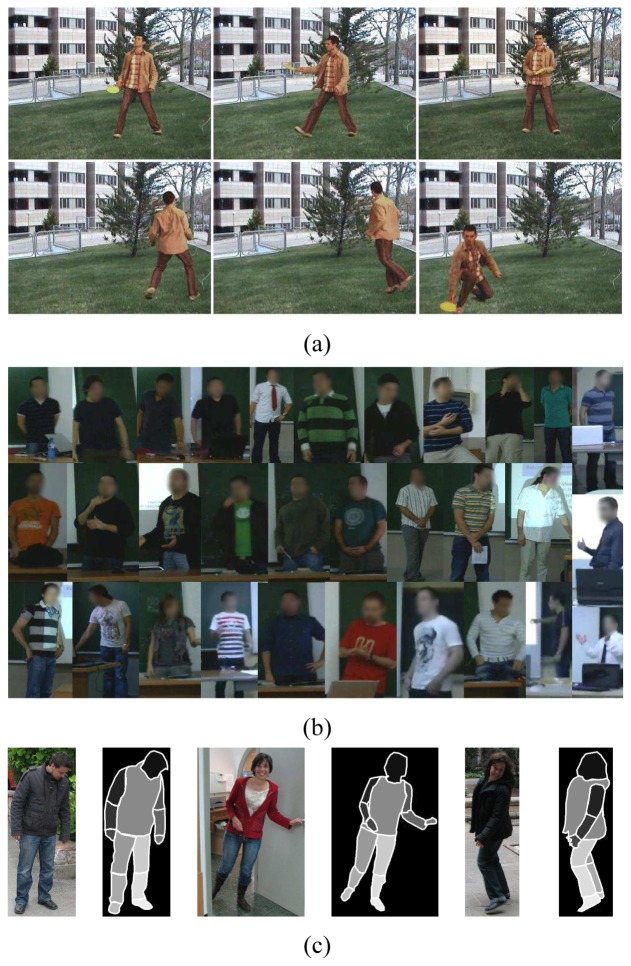
(**a**) Samples of the cVSG corpus and (**b**) UBDataset image sequences, and (**c**) HumanLimb dataset.

We use the public image sequences of the Chroma Video Segmentation Ground Truth (cVSG) [17], a corpus of video sequences and segmentation masks of people. Chroma based techniques have been used to record Foregrounds and Backgrounds separately, being later combined to achieve final video sequences and accurate segmentation masks almost automatically. Some samples of the sequence we have used for testing are shown in Figure 4(a). The sequence has a total of 307 frames. This image sequence includes several critical factors that make segmentation difficult: object textural complexity, object structure, uncovered extent, object size, Foreground and Background velocity, shadows, background textural complexity, Background multimodality, and small camera motion.

As a second database, we have also used a set of 30 videos corresponding to the defense of undergraduate thesis at the University of Barcelona to test the methodology in a different environment (UBDataset). Some samples of this dataset are shown in Figure 4(b).

**Figure 5.**
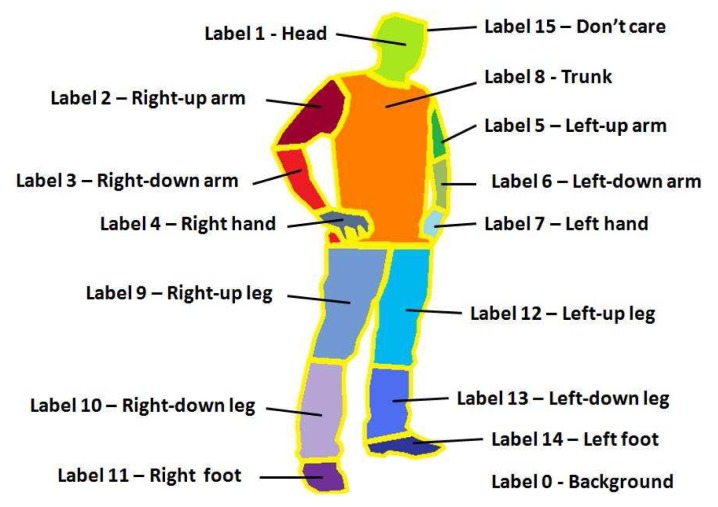
Human Limb dataset labels description.

Moreover, we present the Human Limb dataset, a new dataset composed by 227 images from 25 different people. At each image, 14 different limbs are labeled (see Figure 4(c)), including the “do not care” label between adjacent limbs, as described in Figure 5. Backgrounds are from different real environments with different visual complexity. This dataset is useful for human segmentation, limb detection, and pose recovery purposes [18].

### Methods

We test the classical semi-automatic GrabCut algorithm for human segmentation comparing with the proposed ST-GrabCut algorithm. In the case of GrabCut, we set the number of GMM components *k* = 5 for both foreground and background models. Furthermore, the already trained models used for person and face detectors have been taken from the OpenCV 2.1.

We also test the mesh fitting and body pose recovery methodologies on the obtained segmentations. The body model used for the pose recovery was taken directly from the work of [8].

### Validation measurements

In order to evaluate the robustness of the methodology for human body segmentation, face and pose fitting, we use the ground truth masks of the images to compute the overlapping factor *O* as follows

(13) $$O=\frac{\sum M_{GC}\cap M_{GT}}{\sum M_{GC}\cup M_{GT}}$$

where *M_GC_* and *M_GT_* are the binary masks obtained for spatio-temporal GrabCut segmentation and the ground truth mask, respectively.

### 3.1. Spatio-Tempral GrabCut Segmentation

**Figure 6.**
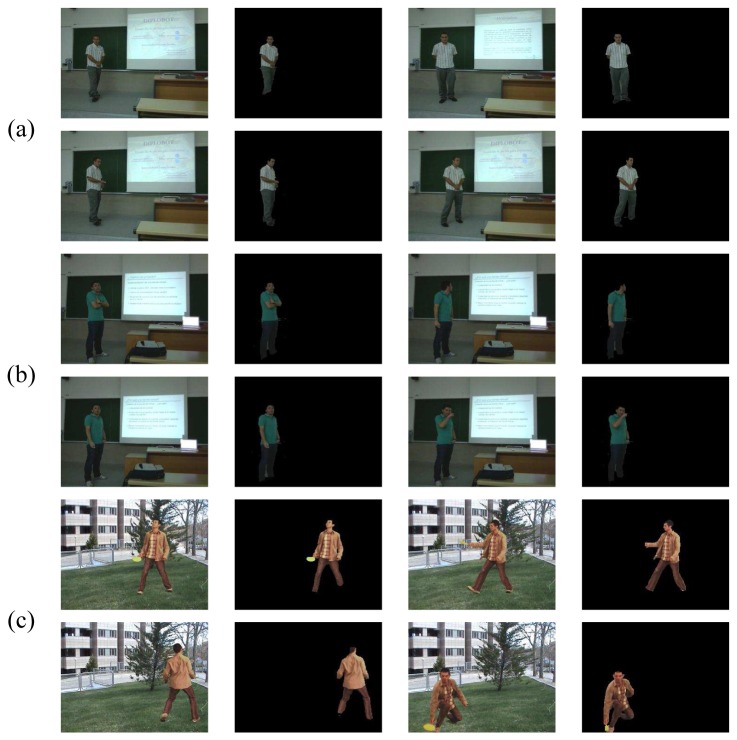
Segmentation examples of (**a**) UBDataset sequence 1, (**b**) UBDataset sequence 2 and (**c**) cVSG sequence.

First, we test the proposed ST-GrabCut segmentation on the sequence from the public cVSG corpus. The results for the different experiments are shown in Table 1. In order to avoid the manual initialization of classical GrabCut algorithm, for all the experiments, seed initialization is performed applying the commented person HOG detection, face detection, and skin color model. First row of Table 1 shows the overlapping performance of Equation (13) applying GrabCut segmentation with *k*-means clustering to design the GMM models. Second row shows the overlapping performance considering the spatial extension of the algorithm introduced by using Mean Shift clustering (Equation (5)) to design the GMM models. One can see a slight improvement when using the second strategy. This is mainly because Mean Shift clustering takes into account spatial information of pixels in clustering time, which better defines contiguous pixels of image to belong to GMM models of foreground and background. Third performance in Table 1 shows the overlapping results adding the temporal extension to the spatial one, considering the morphology refinement based on previous segmentation (Equation (7)). In this case, we obtain near 10% of performance improvement respect the previous result. Finally, last result of Table 1 shows the full-automatic ST-GrabCut segmentation overlapping performance taking into account spatio-temporal coherence, and the segmentation refinement introduced in Equation (6). One can see that it achieves about 25% of performance improvement in relation with the previous best performance. Some segmentation results obtained by the GrabCut algorithm for the cVSG corpus are shown in Figure 6. Note that the ST-GrabCut segmentation is able to robustly segment convex regions. We have also applied the ST-GrabCut segmentation methodology on the image sequences of UBDataset. Some segmentations are shown in Figure 6.

### 3.2. Face Fitting

**Figure 7.**
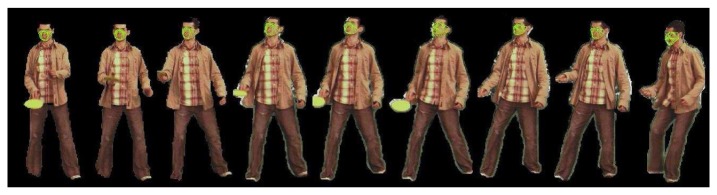
Samples of the segmented cVSG corpus image sequences fitting the different AAM meshes.

In order to measure the robustness of the spatio-temporal AAM mesh fitting methodology, we performed the overlapping analysis of meshes in both un-segmented and segmented image sequence of the public cVSG corpus. Overlapping results are shown in Table 2. One can see that the mesh fitting works fine in unsegmented images, obtaining a final mean overlapping of 89.60%. In this test, we apply HaarCascade face detection implemented and trained by the Open Source Computer Vision library (OpenCv). The face detection method implemented in OpenCV by Rainer Lienhart is very similar to the one published and patented by Paul Viola and Michael Jones, namely called Viola–Jones face detection method [19]. The classifier is trained with a few hundreds of sample views of a frontal face, that are scaled to the same size (20 × 20), and negative examples of the same size. However, note that combining the temporal information of previous fitting and the ST-GrabCut segmentation, the face mesh fitting considerably improves, obtaining a final of 96.36% of overlapping performance. Some example of face fitting using the AAM meshes for different face poses of the cVSG corpus are shown in Figure 7.

To create three AAM models that represent frontal, right and left views, we have created a training set composed by 1,000 images for each view. The images have been extracted from the public database [15]. To build three models we manually put 21 landmarks over 500 images for each view. The landmarks of the remaining 500 images which covers one view, has been placed by a semi-automatic process, applying AAM with the set learnt and manually correcting. Finally, we align every resulting mesh and we obtain the mean for each model. As the head pose classifier, to classify the spatial mesh configuration in 5 head poses, we have labeled manually the class of the mesh obtained applying the closest AAM model. Every spatial mesh configuration is represented by the 20 most representative eigenvectors. The training set is formed by 5,000 images from the public database [15]. Finally, we have tested the classification of the five face poses on the cVSG corpus, obtaining the percentage of frames of the subject at each pose. The obtained percentages are shown in Table 3.

### 3.3. Body Limbs Recovery

**Figure 8.**
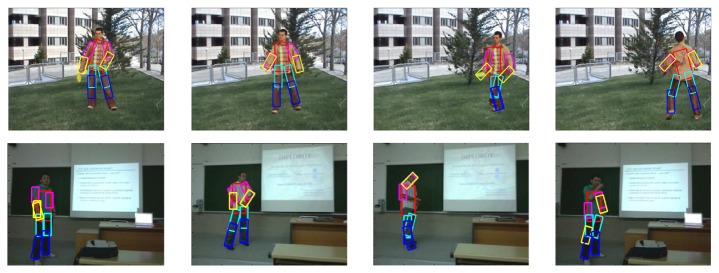
Pose recovery results in cVSG sequence.

Finally, we combine the previous segmentation and face fitting with a full body pose recovery [8]. In order to show the benefit of applying previous ST-GrabCut segmentation, we perform the overlapping performance of full pose recovery with and without human segmentation, always within the bounding box obtained from HOG person detection. Results are shown in Table 4. One can see that pose recovery considerably increases its performance when reducing the region of search based on ST-GrabCut segmentation. Some examples of pose recovery within the human segmentation regions for cVSG corpus and UBdataset are shown in Figure 8. One can see that in most of the cases body limbs are correctly detected. Only in some situations, occlusions or changes in body appearance can produce a wrong limb fitting.

**Figure 9.**
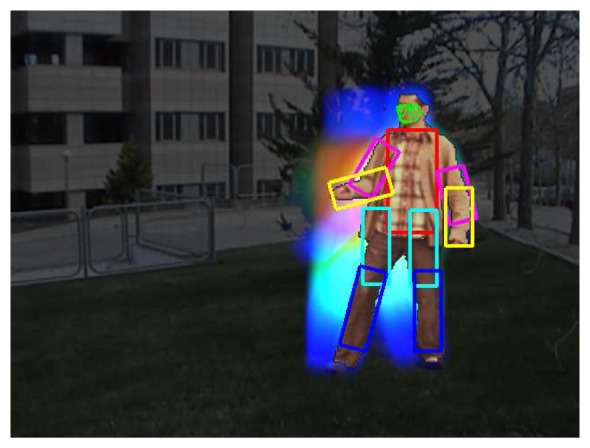
Application of the whole framework (pose and face recovery) on an image sequence.

In Figure 9 we show the application of the whole framework to perform temporal tracking, segmentation and full face and pose recovery. The colors correspond to the body limbs. The colors increase in intensity based on the instant of time of its detection. One can see the robust detection and temporal coherence based on the smooth displacement of face and limb detections.

### 3.4. Human Limb Data Set

**Figure 10.**
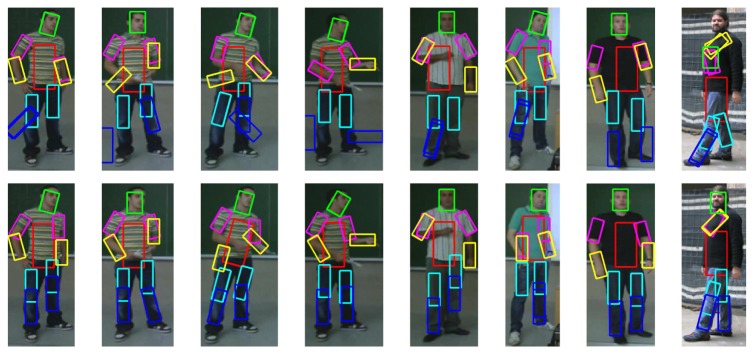
Human Limb dataset results. Up row: limb recovery without ST-GrabCut segmentation. Down row: limb recovery with ST-GrabCut segmentation.

**Figure 11.**
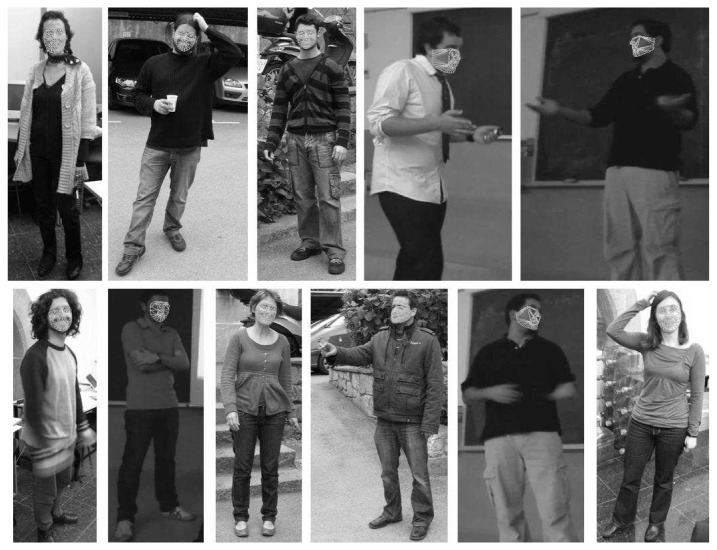
Application of face recovery on human body limb dataset.

In this last experiment, we test our methodology on the presented Human Limb dataset. From the 14 total limb annotations, we grouped them into six categories: trunk, up-arms, up-legs, low-arms, low-legs, and head, and we tested the full pose recovery framework. In this case, we tested the body limb recovery with and without applying the ST-GrabCut segmentation, and computed three different overlapping measures: (1) %, which corresponds to the overlapping percentage defined in Equation (13); (2) wins, which corresponds to the number of Limb regions with higher overlapping comparing both strategies; (3) match, which corresponds to the number of limb recoveries with overlapping superior to 0.6. The results are shown in Table 5. One can see that because of the reduced region where the subjects appear, in most cases there is no significant difference applying the limb recovery procedure with or without previous segmentation. Moreover, the segmentation algorithm is not working at maximum performance due to the same reason, since very small background regions are present in the images, and thus the background color model is quite poor. Furthermore, in this dataset we are working with images, not videos, and for this reason we cannot include the temporal extension in our ST-GrabCut algotithm for this experiment. On the other hand, looking at the mean average overlapping in the last column of the table, one can see that ST-GrabCut improves for all overlapping measures the final limb overlapping. In particular, in the case of the Low-legs recovery is when a more clear improvement appears using ST-GrabCut segmentation. The part of the image corresponding to Low-legs is where more background influence exists, and thus the limb recovery has the highest confusion. However, as ST-GrabCut is able to properly segment the concave regions of the Low-legs regions, a significant improvement is obtained when applying the limb recovery methodology. Some results are illustrated on the images of Figure 10, where the images on the bottom correspond to the improvements obtained using the ST-GrabCut algorithm. Finally, Figure 11 show examples of the face fitting methodology applied on the human body limb dataset.

## 4. Conclusions

In this paper, we presented an evolution of the semi-automatic GrabCut algorithm for dealing with the problem of human segmentation in image sequences. The new full-automatic ST-GrabCut algorithm uses a HOG-based person detector, face detection, and skin color model to initialize GrabCut seeds. Spatial coherence is introduced via Mean Shift clustering, and temporal coherence is considered based on the historical of Gaussian Mixture Models. The segmentation procedure is combined with Shape and Active Appearance models to perform full face and pose recovery.

This general and full-automatic human segmentation, pose recovery, and tracking methodology showed higher performance than classical approaches in public image sequences and a novel Human Limb dataset from uncontrolled environments, which makes it useful for general human face and gesture analysis applications.

One of the limitations of the method is that it depends on the initialization of the ST-GrabCut algorithm, which basically depends on the person and face detectors. Initially, we wait until at least one bounding box is returned by the person detector. This is a critical point, since we will trust the first detection and start segmenting with this hypothesis. In contrast, there is no problem if a further detection is missed, since we initialize the mask with the previous detection (temporal extension). Moreover, due to its sequential application, false seed labeling can accumulate segmentation errors along the video sequence. As the next step, we plan to extend the limb recovery approach so that more complex poses and gestures can be recognized, and feed a gesture recognition system [20] with the temporal aggregation of the recovered poses along the sequence in order to look for motion patterns of the limbs.

As a future work, the algorithm could be extended in order to segment sequences with more than one person present in the images, since our current method only segments one subject in the scene.

## Figures and Tables

**Table 1.** GrabCut and ST-GrabCut Segmentation results on cVSG corpus.

Approach	Mean overlapping
GrabCut	0.5356
Spatial extension	0.5424
Temporal extension	0.6229
ST-GrabCut	0.8747

**Table 2.** AAM mesh fitting on original images and segmented images of the cVSG corpus.

Approach	Mean overlapping
Mesh fitting without segmentation	0.8960
ST-Grabcut & Temporal mesh fitting	0.9636

**Table 3.** Face pose percentages on the cVSG corpus.

Face view	System classification	Real classification
Left view	0.1300	0.1211
Near Left view	0.1470	0.1347
Frontal view	0.2940	0.3037
Near Right view	0.1650	0.1813
Right view	0.2340	0.2590

**Table 4.** Overlapping of body limbs based on ground truth masks.

Approach	Mean overlapping
Limb recovery without segmentation	0.7919
ST-Grabcut & Limb recovery	0.8760

**Table 5.** Overlapping percentages between body parts (intersection over union), wins (comparing the highest overlapping with and without segmentation), and matching (considering only overlapping greater than 0.6).

		**Trunk**	**Up-arms**	**Up-legs**	**Low-arms**	**Low-legs**	**Head**	**Mean**
%	**No segmentation**	0.58	0.53	0.59	0.50	0.48	0.67	0.56
	STGrabCut*	0.58	0.53	0.58	0.50	0.56	0.67	**0.57**

Wins	**No segmentation**	106	104	108	109	68	120	102.5
	STGrabCut*	121	123	119	118	159	107	**124.5**

Match	**No segmentation**	133	127	130	121	108	155	129
	STGrabCut*	125	125	128	117	126	157	**129.66**

* STGrabCut was used without taking into account temporal information.
